# Improving test properties for neonatal cystic fibrosis screening in the Netherlands before the nationwide start by May 1st 2011

**DOI:** 10.1007/s10545-012-9452-7

**Published:** 2012-02-03

**Authors:** Martina C. Cornel, Johan J. P. Gille, J. Gerard Loeber, Annette M. M. Vernooij-van Langen, Jeannette Dankert-Roelse, Piet A. Bolhuis

**Affiliations:** 1 Clinical Genetics, VU University Medical Centre, Amsterdam, The Netherlands; 2 Centre for Society and Genomics, Nijmegen, The Netherlands; 3 Centre for Medical Systems Biology, Leiden, The Netherlands; 4 Laboratory for Infectious Diseases and Perinatal Screening, National Institute for Public Health, Bilthoven, The Netherlands; 5 Department of Pediatrics, Atrium medical centre Parkstad, Heerlen, The Netherlands; 6 Forum Biotechnology and Genetics, The Hague, The Netherlands; 7 EMGO/Clinical Genetics, BS7 D423, VU University Medical Centre, PO Box 7057, 1007 MB Amsterdam, The Netherlands

## Abstract

When new technical possibilities arise in health care, often attunement is needed between different actors from the perspectives of research, health care providers, patients, ethics and policy. For cystic fibrosis (CF) such a process of attunement in the Netherlands started in a committee of the Health Council on neonatal screening in 2005. In the balancing of pros and cons according to Wilson and Jungner criteria, the advantages for the CF patient were considered clear, even though CF remains a severe health problem with treatment. Nevertheless, screening was not started then, mainly since the specificity of the tests available at that time was considered too low. Many healthy infants would have been referred for sweat testing and much uncertainty would arise in their parents. Also the limited sensitivity for immigrants and the detection of less severe phenotypes and carriers were considered problematic. The Health Council recommended a pilot screening project which was subsequently performed in some provinces, leading to a 4-step protocol: IRT, PAP, screening for a *CFTR* mutation panel, and sequencing of the *CFTR* gene. This would lead to the identification of 23 cases of classical CF, two infants with less severe forms and 12 carriers per year in the Netherlands. Thus many CF patients can be diagnosed early, while limiting the number of referrals, the number of infants with less severe forms diagnosed and the number of carriers identified. Technical solutions were found to limit the ethical problems. A nationwide program using this four step protocol started by 1 May 2011.

## Introduction

Cystic fibrosis (CF), also known as mucoviscidosis, is an autosomal recessive condition causing chronic obstructive pulmonary disease, nutritional problems, pancreatic fibrosis, hepatic fibrosis, diabetes and azoospermia. The life expectancy of cystic fibrosis patients has increased in the last decades: in 1960-1970 50% of patients in the Netherlands reached the age of 8 years (Dankert-Roelse et al. 1986), in 1983 the average life expectancy was 23 years, and nowadays it is 35–40 years. Neonatal screening has long been expected to contribute to a longer and healthier life in CF patients. In 1978 a Dutch study reported on the results of a pilot to detect albumin in meconium of 68,000 neonates (Ten Kate 1978). The positive predictive value of the test turned out to be only 3.39%, and thus it was concluded that screening might have more disadvantages than not screening. In the same period several other countries did start CF screening (Grosse et al. 2006). Australian colleagues started detecting IRT in blood samples, and showed that hospital admissions in the first two years of life were reduced in screened patients (a mean of 3.9 days in the first two years of life in screened vs. 27.3 days in unscreened patients) (Wilcken and Chalmers 1985). An important question is whether life expectancy is increased by screening. No such increase was found in an early randomized controlled trial in Wisconsin, since screened patients who met in the CF centre unfortunately had earlier colonization with *Pseudomonas aeruginosa* (Southern et al. 2009). This has influenced the decision making in the Netherlands, even after later studies showed better pulmonary outcomes, nutritional benefits and reduced CF-related mortality risk to approximately 10 years of age after CF neonatal screening (Grosse et al. 2006; Southern et al. 2009).

This paper describes the process of decision making in the Netherlands to advise against cystic fibrosis newborn screening in 2005, the improvements of test properties until it was considered appropriate to be included in the program and nationwide screening was started by 1 May 2011.

## Insufficient specificity?

High throughput techniques made neonatal screening for many diseases technically possible after the year 2000. New technical possibilities need attunement by all parties involved, such as scientists in laboratories and clinics, physicians and other professionals in (public) health care, patients (organizations) and regulatory, advisory and governmental agencies (Achterbergh et al. 2007).

In The Netherlands the Health Council installed a commission to study the pros and cons of potential neonatal screening possibilities in 2005, from the point of view of a diversity of backgrounds, including paediatrics, genetics, ethics and law (Health Council of the Netherlands. Neonatal Screening. The Hague: Health Council of the Netherlands 2005). The report led to the extension of the program to 17 disorders, not including cystic fibrosis. The fact that cystic fibrosis treatment confers a substantial health gain was considered beyond dispute. The question was, however, how much screening immediately after birth contributes to this health gain? The committee considered cystic fibrosis a “borderline case”, as the evidence of health gain was less compelling than in the case of disorders such as phenylketonuria and hypothyroidism. The advantages of neonatal screening for cystic fibrosis, namely a better nutritional status, prevention of an often protracted and aggravating diagnostic process and a decrease in the number of incidents of sickness and hospital admissions were considered clear (Health Council of the Netherlands. Neonatal Screening. The Hague: Health Council of the Netherlands 2005). The imperfections of the screening were however the main reason not to advise to include cystic fibrosis in the neonatal screening program, but rather to undertake research into better screening methods. In the 2005 Health Council report (Health Council of the Netherlands. Neonatal Screening. The Hague: Health Council of the Netherlands 2005), an IRT/DNA screening program was estimated to lead to the diagnosis of 50-60 patients per year, 600 infants referred for sweat test, and 400 heterozygotes diagnosed (carriers of CF). The Health Council recommended to include cystic fibrosis in neonatal heel prick screening as soon as a test method would become available with a high specificity (as low specificity leads to a great number of clinical investigation of unaffected neonates). A study to improve the test properties of cystic fibrosis neonatal screening was indeed initiated: CHOPIN (Cystic fibrosis heelprick screening in a newborn population in the Netherlands).

## CHOPIN pilot study

A pilot study to improve test properties was performed in 2008 and 2009 in four provinces of the Netherlands: Utrecht, Gelderland, Noord-Brabant and Limburg. The study was led by two of us (JD and AV). Two screening strategies were compared: measuring the serum concentration of immunoreactive trypsinogen (IRT) followed by pancreatitis associated protein (PAP) (Sarles et al. 2005) vs IRT followed by a DNA test panel of 35 mutations in the cystic fibrosis transmembrane regulator (*CFTR*) gene^1^ and, if one *CFTR* mutation was found, extended gene analysis (EGA; DNA sequencing of all coding exons and intron/exon boundaries of the *CFTR* gene). In 2011 the results of the study have been accepted for publication (Vernooij-van Langen et al. 2011), but a short summary of the results was already presented in a 2010 Health Council report (Health Council of the Netherlands. Neonatal screening for cystic fibrosis. The Hague: Health Council of the Netherlands 2010). Apart from the two strategies followed in the pilot, a third scenario has been calculated, combining the two strategies. IRT/PAP leads to a relatively large number of referrals (119 as compared to 20 in the IRT/*CFTR* mutation panel/EGA strategy). The specificity of IRT/*CFTR* mutation panel/EGA is higher but this strategy will also identify carriers and less severe forms of CF, as can be seen in Table 1. PAP as a second step added to the IRT/*CFTR* mutation panel/EGA strategy will reduce the number of carriers and less severe forms. Thus IRT/PAP/*CFTR* mutation panel/EGA combines the best screening test properties.

**Table 1:** Results heel prick screening (CHOPIN study) of 72 874 newborns (Health Council of the Netherlands. Neonatal screening for cystic fibrosis. The Hague: Health Council of the Netherlands 2010)

	IRT ≥ 50 μg/l and PAP ≥ 1.8 μg/l* or IRT ≥ 100 μg/l and PAP ≥ 1.0 μg/l	IRT ≥ 50 μg/l followed by DNA-EGA	IRT-PAP-DNA-EGA (calculated results)
Abnormal results	119	20	12
Classical CF	10	10	10
Non-classical CF	0	9	2
Carriers	0	89	5

* Up to 2010 Dynabio based its PAP-kit calibrators on the assumption that a 3 mm-punch contains 5 μl blood. The CDC Newborn Screening Annual summary reports however show that this volume is in fact 3 μl. (Adam et al. 2000). Hence, Dynabio issued a statement in March 2011 via the website of the International Society for Neonatal Screening (Dagorn 2011) that all concentrations should be corrected by multiplying them by a factor of 5/3.

## From pilot to national program

**Fig. 1:**
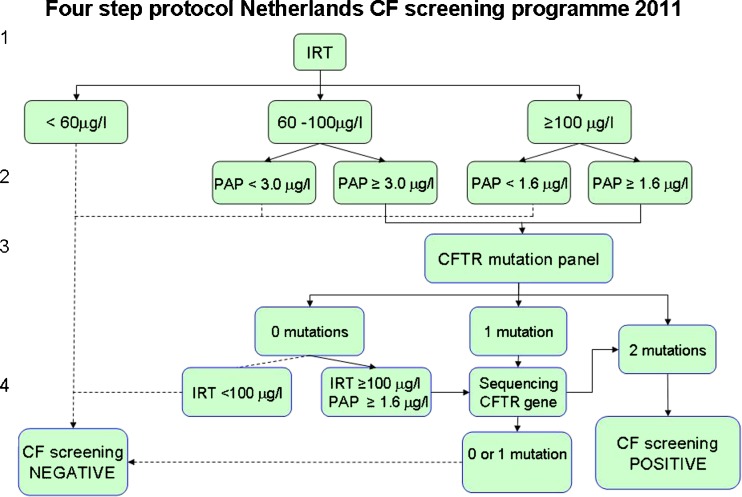
Four step screening protocol Netherlands CF screening programme 2011

The Health Council again asked a committee to advise on CF newborn screening after the CHOPIN study had been finished (Health Council of the Netherlands. Neonatal screening for cystic fibrosis. The Hague: Health Council of the Netherlands 2010). Apart from the strategies above, the committee discussed the IRT limit (≥50 μg/l = the 2.43% highest concentrations vs. ≥60 μg/l = the highest 1.03%), and considered that an increase of the limit for IRT to 60 μg/l produces a sharp drop in the number of non-classical CF patients identified by the screening. Also, in case of IRT ≥ 100 μg/l and PAP ≥ 1.6 μg/l, sequencing of the *CFTR* gene was included even if none of the 35 mutations was present, since then the risk of a rare mutation is relatively high. A four step protocol was proposed for the national program (Fig. 1):

1. IRT <60 μg/l: negative, otherwise
2. PAP < 3.0 μg/l if IRT 60–100 μg/l or < 1.6 μg/l if IRT ≥100 μg/l : negative, otherwise
3. *CFTR* mutation panel including all common *CFTR* mutations in the Netherlands. Proceed to next step if 1 mutation or IRT ≥ 100 μg/l and PAP ≥ 1.6 μg/l :
4. DNA sequencing of all coding exons and intron/exon boundaries of the *CFTR* gene

The PAP cut off levels are identical to the ones earlier reported by Sarles et al. (2005). However, recently the manufacturer of the PAP kits has recognized a systematic error in his calibration procedure. This four step protocol uses the corrected values (Dagorn 2011).

This protocol was expected to identify 25 CF patients on an annual basis, additional to four infants already diagnosed because of meconium ileus (Health Council of the Netherlands. Neonatal screening for cystic fibrosis. The Hague: Health Council of the Netherlands 2010). Only 12 carriers of CF would be identified. Out of the 25 CF patients 23 were expected to have only mutations from the panel and two to be identified by extended mutation analysis. This protocol was proposed by the Health Council for national implementation, and accepted by the ministry of Health.

After the start of the four step protocol on a nationwide basis by 1 May 2011, in the first 3 months 11 CF patients were identified: eight because of two mutations in the panel and three with one mutation in the panel and one identified by sequencing the *CFTR* gene, as preliminary reported by the laboratories involved.

Due to the screening program CF patients will have a better nutritional status. An often protracted and aggravating diagnostic process is prevented, a decrease in hospital admissions anticipated and other benefits seem likely. The specificity is >99.99%.

## Sensitivity

While the specificity of CF neonatal screening was the main test property to be improved, according to the Health Council advice in 2005, also the sensitivity needed improvement. In view of the large number of rare mutations not included in the *CFTR* mutation panel a failsafe procedure was built in the protocol. Earlier a failsafe procedure was performed in Massachusetts using a multiple CF mutation screen, which added somewhat to the sensitivity, however at the cost of increased referrals and carrier identification (Comeau et al. 2004). In the current Netherlands procedure, the *CFTR* gene is sequenced in the last step. The procedure is especially important for infants with migrant ancestors, since the sensitivity of the *CFTR* mutation panel was only 44% for Turkish migrants and 69% for North African migrants in Europe (Health Council of the Netherlands. Neonatal screening for cystic fibrosis. The Hague: Health Council of the Netherlands 2010; Lakeman et al. 2008). The CF carrier frequency in Dutch inhabitants of Turkish and North African ancestry can only be roughly approximated, but is estimated to be around 1 in 50. The annual number of neonates with CF in these population groups is estimated to be 1-2. The failsafe procedure consisted of sequencing the *CFTR* gene in case of an IRT concentration of ≥100 μg/l and PAP ≥ 1,6 μg/l, also if no mutation was identified using the *CFTR* mutation panel. It is hard to estimate the sensitivity, but this is not expected to be 100%, due to IRT and PAP. The consequence of pursuing an extremely high specificity is in general a less optimal sensitivity. Based on the CHOPIN study the sensitivity might be ±95%.

## Is carrier status information an additional benefit?

While the goal of neonatal screening is to identify infants with two *CFTR* mutations, that lead to the classical form of cystic fibrosis, some infants will be diagnosed as CF carriers, having only one *CFTR* mutation. If the newborn child is a carrier, then it follows that one, or both, parents (and possibly other children) are carriers (Health Council of the Netherlands. Neonatal screening for cystic fibrosis. The Hague: Health Council of the Netherlands 2010). As this may be relevant information to the parents in connection with future family planning, they are informed of this secondary finding. Parents in this situation can be referred for genetic counselling. At the moment the heel prick is performed, parents can opt-out of receiving carrier status information. To allow parents to make an informed decision on (not) receiving carrier status information, the parents should be alerted to these possible outcomes prior to screening. Thus the information and counselling given to parents during pregnancy becomes more complex. After birth, information of this kind can, in practice, give rise to misunderstandings with regard to the health of the carriers.

In theory, genetic screening can be performed in different phases of life: before conception, during pregnancy, in the newborn or later in life. Before pregnancy (in preconceptional screening) more reproductive options exist, such as choosing not to have children (or adopting a child), preimplantation genetic diagnosis (embryo selection), prenatal diagnosis and termination of affected foetuses, choosing a different partner or using donor gametes (e.g. artificial insemination by donor sperm). However, in most countries preconceptional screening for cystic fibrosis is not available in health care.

## Increase of CF risk

**Fig. 2:**
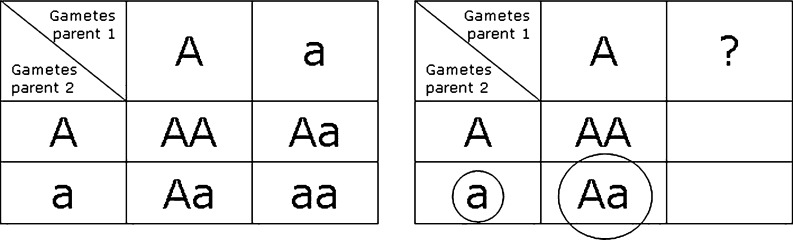
Punnett squares for an autosomal recessive disorder and for parents of a CF carrier infant. Left: Punnett square for autosomal recessive disorder. Right: Punnett square for parents of CF carrier infant (large circle), one of which carries a CF allele (small circle). If both parents do not have the CF phenotype, this carrier parent must also have a normal *CFTR* allele (A). The risk for the unknown allele to carry a *CFTR* mutation is the allele frequency of a. The risk to have a child with CF is the ¼ x allele frequency

The Punnett squares in Fig. 2 show the information that can be derived from the fact that a child of healthy parents is a CF carrier. If the child is a carrier, at least one parent is carrier as well. If none of the parents have the CF phenotype, the carrier parent must also have a normal *CFTR* gene. If the carrier frequency in a population is 1:30, the allele frequency is 1:60, and for each next pregnancy the risk of a CF affected infant is 1/4x1/60 = 1/240, much higher than the birth prevalence would be (1/3600). A CF carrier test in the parents might provide certainty. However, as the 2005 Health Council report (Health Council of the Netherlands. Neonatal Screening. The Hague: Health Council of the Netherlands 2005) mentions, “… it is not always possible to determine for certain whether only one parent is a carrier (as is the case with, for example, cystic fibrosis, where not all mutations are known).” This holds true when CF testing is based on a *CFTR* mutation panel. Sequencing of the *CFTR* genes of the parents will reduce the residual risk to a figure far lower than the population risk.

## Future research

After the decision to start a national CF screening program, there are several questions that remain to be answered. Further evaluation of the sensitivity and the specificity achieved in the current four steps is needed. In an a different IRT/PAP study published last year the IRT/PAP strategy according to Sarles et al. (2005) showed some limitations, since not all CF patients with severe forms were found: PAP values of 3 out of 13 newborns with CF (23%) were equal to or below the cut-off level (Sommerburg et al. 2010). Further evaluation might lead to changes of the cut-off levels.

## Conclusion

The national neonatal CF screening program in the Netherlands that is in place since 1 May 2011, has a specificity >99.99%. The sensitivity is expected to be ±95%, but further evaluation is needed. Carrier status information is provided to only a small number, if parents do not opt out. A clinical geneticist can provide counselling to carriers upon request.

## Abbreviations

CF: Cystic fibrosis
CFTR: Cystic fibrosis transmembrane regulator
CHOPIN: Cystic fibrosis heelprick screening in a newborn population in the Netherlands
EGA: Extended gene analysis
IRT: Immunoreactive trypsinogen
PAP: Pancreatitis associated protein

## Footnotes

1 Using the LiPA test (INNO-LiPA *CFTR* 19 en INNO-LiPA *CFTR* 17+Tn; Innogenetics, Gent, Belgium) the following *CFTR* mutations can be detected: exon 2-3del (21 kb), 394delTT, E60X, G85E, R117H, 621+1G>T, 711+1G>T, 711+5G>A, 1078delT, R334W, R347P, A455E, I507del, F508del, 1717-1G>A, G542X, G551D, Q552X, R553X, R560T, 1898+1G>A, 2143delT, 2183AA>G, 2184delA, 2789+5G>A, 3120+1G>A, 3199del6, 3272-26A>G, 3659delC, R1162X, 3849+10kbC>T, 3905insT, S1251N, W1282X en N1303K. This test also identifies the CFTR polymorphism Tn in intron 8 which is important in cases where the mutation R117H is detected. Mutation I148T, which is still part of this test, was ignored since this mutation is not considered disease-causing anymore.
